# [Retracted] miR-124-3p inhibits the viability and motility of glioblastoma multiforme by targeting RhoG

**DOI:** 10.3892/ijmm.2026.5743

**Published:** 2026-01-26

**Authors:** Shan Cai, Chao-Jia Shi, Jian-Xiang Lu, Yan-Pei Wang, Tian Yuan, Xiang-Peng Wang

Int J Mol Med 47: 69, 2021; DOI: 10.3892/ijmm.2021.4902

Following the publication of this paper, a concerned reader drew to the Editor's attention that, according to a study published by Kristin Entrop and colleagues in the journal *Cell Death & Disease* in 2024, an unspecific anti-Bax antibody (sc-7480) had been used in the above study, which was shown to bind to an unrelated protein at the correct molecular weight (20-25 kDa). Furthermore, it also came to light that, for the cell migration and invasion assay data shown in Fig. 8B on p. 10, at least four pairs of data panels were shown to feature overlapping sections, such that data which were intended to show the results of differently performed experiments had apparently been derived from a smaller number of original sources.

After having performed an independent review of the data in the Editorial Office, the Editor of *International Journal of Molecular Medicine* has decided that this paper should be retracted from the Journal on account of a lack of confidence in the presented data. The authors were asked for an explanation to account for these concerns, but the Editorial Office did not receive a reply. The Editor apologizes to the readership for any inconvenience caused.

